# Evaluating *Caenorhabditis elegans* as a Toxicity Model for Reuptake Inhibitors

**DOI:** 10.17912/micropub.biology.002053

**Published:** 2026-07-03

**Authors:** Christopher E. Hernandez, Alexandra Van Stone, Christopher So

**Affiliations:** 1 College of Pharmacy, Roseman University of Health Sciences, Henderson, Nevada, United States; 2 College of Graduate Studies, Roseman University of the Health Sciences, Henderson, Nevada, USA

## Abstract

Drug toxicity assessment is important for drug development. Here, we evaluated whether the invertebrate model *Caenorhabditis elegans* can be used to assess the toxicity of selective serotonin reuptake inhibitors (SSRIs) and serotonin–norepinephrine reuptake inhibitors (SNRIs). Drug-induced non-responsiveness served as a functional measure of toxicity. Overall, responses did not consistently parallel those of mammals, although select trends were conserved. Escitalopram showed greater toxicity than citalopram, duloxetine was more toxic than milnacipran, and desvenlafaxine, but not venlafaxine, produced toxicity. These results suggest that *C. elegans* cannot replace mammalian testing, but may serve as a rapid and low-cost prescreening model.

**Figure 1. Concentration–Response Assessment of SSRI and SNRI Toxicity in Caenorhabditis elegans:** Figure 1: Response of *C. elegans* to increasing concentrations (100 nM-4 mM) of select SSRIs and SNRIs with the original stock solutions initially prepared in different solvents. A) Responsiveness to SSRIs with the original stock solutions initially prepared in water (n=4). B) Responsiveness to citalopram or escitalopram with stock solutions initially prepared in DMSO (n=4) or ethanol (n=4). C) Responsiveness to SNRIs with stock solutions initially prepared in water (n=4). D) Responsiveness to venlafaxine and desvenlafaxine with the original stock solutions prepared in DMSO (n=4). &nbsp;

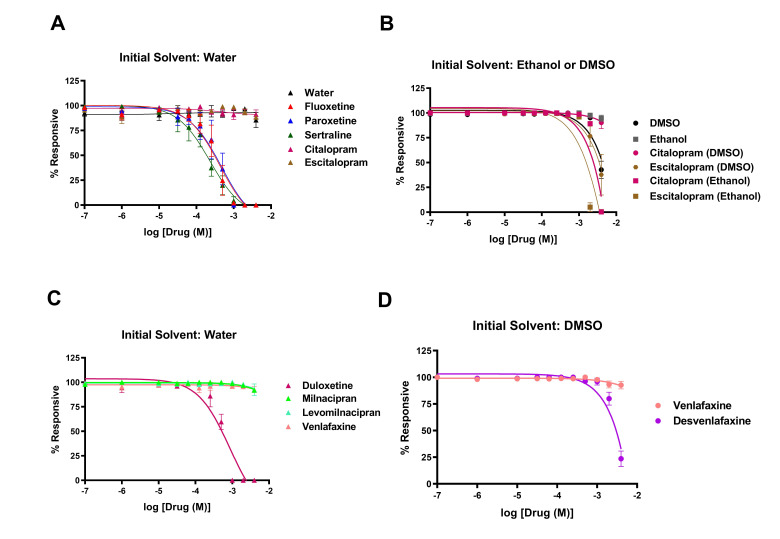



## Description

One underexploited avenue for *Caenorhabditis elegans* is drug testing and toxicity assessment. Although some studies have used *C. elegans* for testing drug toxicity after the fact (Hunt, 2017), these animals have rarely been part of the normal drug development procedures (Letizia et al., 2018), even though medications have targets within *C. elegans* (Hobert, 2013). However, with the cost of drug testing in mammalian models very high (Doke and Dhawale, 2015), potentially the nonmammalian models can offer a cheaper alternative (Khabib et al., 2022) that will not replace the required animal tests prior to drug approval, but may give guidance as to the drug design and development process.

This study evaluates the toxicity of selective serotonin reuptake inhibitors (SSRIs) and serotonin-norepinephrine reuptake inhibitors (SNRIs) in C. elegans . These drugs were selected because they are prescribed for mental health disorders (Machado and Einarson, 2010) and are generally considered safe, although some side effects are more noticeable than others (Wang et al., 2018). Furthermore, their toxicities, prior to drug approval for clinical use, were tested in rabbits, mice, and rats (Byrd and Markham, 1994). Limited studies have evaluated the toxicological effects of these medications in *C. elegans ,* even though these drugs target the serotonin transporter *mod-5* , which is analogous to that found in mammals (Ranganathan et al., 2001; Yu et al., 2023). Furthermore, these animals contain a serotonergic pathway that is responsive to antidepressants (Weinshenker et al., 1995). Therefore, it would be of interest to assess their drug-induced toxicities in nematodes and explore if the toxic effects found within this organism would have been predictive of the toxicity found in the mammalian model organisms.

First, we assessed the toxicity of SSRIs by first preparing their original stock solution in water, followed by serial dilutions in water (FIG 1A). We note that *C. elegans* drug exposures are typically performed in isotonic saline to avoid subjecting animals to osmotic stress, and that our resulting data are likely influenced by these hypotonic conditions. We observed a loss of responsiveness, which may stem from drug toxicity, for fluoxetine, paroxetine, and sertraline (Fluoxetine LC _50_ : 0.51 + 0.15 mM; paroxetine LC _50_ : 2.1 + 0.9 mM; and sertraline LC _50_ : 0.28 + 0.0001 mM). This, however, was not observed for citalopram and escitalopram. This rank order of LC _50_ does not correlate with either reported mouse or rat LC _50_ , where sertraline is least toxic (Davies and Kluwe, 1998). For citalopram and escitalopram, we also repeated this experiment by initially preparing their original stock solution in DMSO or ethanol, followed by serial dilutions in water (FIG 1B). When prepared first in DMSO, citalopram demonstrated no toxic effects, but escitalopram showed toxicity at the highest concentration (escitalopram LC _50_ : 708 + 29 mM). This observed effect with escitalopram, but not citalopram, when first prepared in DMSO, is consistent with the greater pharmacological activity of escitalopram reported in mammalian models (Sánchez et al., 2004). This effect was observed when the original stock of citalopram and escitalopram was first prepared in ethanol (Escitalopram LC _50_ : 11.17 + 0.48 mM) (FIG 1B).

Next, we assessed the toxicity of SNRIs when their original chemical stocks were initially prepared in water, followed by serial dilutions in water (FIG 1C). We observed toxicity for only duloxetine (Duloxetine LC _50_ : 0.88 + 0.17 mM). This observation differs from that observed in rats, where LC _50s_ are observed for all SNRIs. However, the fact that duloxetine does demonstrate an LC _50_ in our studies suggests duloxetine may contribute to this through an alternate target in *C. elegans* . For venlafaxine, we also prepared its original drug stock in DMSO followed by serial dilutions in water. Like that observed when prepared in water, no toxic effects were observed (FIG 1D). On the other hand, when its active metabolite desvenlafaxine was used in this experiment, we observed toxicity (Desvenlafaxine LC _50_ : 1.24 + 0.15 M). This suggests that *C. elegans* may have limited metabolic capacity to convert venlafaxine to its active metabolite.

These findings highlight the limitations in using *C. elegans* viability and non-responsiveness alone to predict mammalian toxicity of reuptake inhibitors, as observed with the high concentrations needed to render the animals unresponsive. What may also contribute to this is that the route of absorption of the *C. elegans* of these medications is different from mammals, with the cuticle forming a barrier to limit drug absorption (Xiong et al., 2017). Other indicators of *C. elegans* output may be better gauges of drug toxicology, such as worm growth, development, feeding rate, and motility. These endpoints may provide more informative measures of drug-induced dysfunction collectively alongside viability than observing this variable alone. Notably, drug potency varies depending on the solvent used to initially dissolve the drug. This indicates that the method of original stock preparation can influence apparent toxicity in *C. elegans* assays. Altogether, these results suggest that while *C. elegans* may not replace mammalian toxicology models, it may serve as a rapid, low-cost prescreening tool to prioritize compounds before vertebrate testing.

## Methods

The original drug stocks were prepared first by either dissolving in water, DMSO, or ethanol, establishing an original stock concentration between 20 and 50 mM. From these original stock concentrations, the drugs were then diluted to 4 mM in water. This was performed to minimize the issue of solubility of these drugs even in diluted concentrations. Thereafter, serial dilutions were performed in water on a 96-well plate. All experimental groups, including vehicle controls, were subjected to identical exposure conditions and durations. The volume was 100 μl of water per well. For *C. elegans* maintenance, animals were maintained at 20 °C. The *C. elegans* N2 strain was synchronized by bleaching, then allowed to grow on NGM plates with OP50 for 48 hours, reaching the L4 stage. Thereafter, animals were collected by aspiration in water, and 50-80 animals were transferred into a well within a 96-well plate with concentrations of drugs or vehicles. The *C. elegans* were then incubated in select concentrations for 24 hours at 20 °C. The next day, each well with various concentrations of drugs or vehicles was prodded with a platinum rod, and the number of responsive and nonresponsive animals was scored. These data were then expressed as % responsive (number of responsive/total). These data were then analyzed by Graphpad Prism, and LC _50_ s were expressed as LC _50_ + standard error of the mean.

## Reagents

Fluoxetine, duloxetine, and venlafaxine were purchased from TCI Chemicals Inc (Portland, OR). Citalopram and escitalopram were purchased from Thermo Fisher (Waltham, MA). Desvenlafaxine, milnacipran, and levomilnacipran were purchased from Selleckchem (Houston, TX). Paroxetine and sertraline were purchased from Matrix Scientific (Elgin, SC).
